# Mushroom data creation, curation, and simulation to support classification tasks

**DOI:** 10.1038/s41598-021-87602-3

**Published:** 2021-04-14

**Authors:** Dennis Wagner, Dominik Heider, Georges Hattab

**Affiliations:** Department of Mathematics and Computer Science, University of Marburg, 35043 Marburg, Germany

**Keywords:** Computational models, Data integration, Data mining, Data processing, Machine learning, Quality control, Classification and taxonomy, Scientific data

## Abstract

Predicting if a set of mushrooms is edible or not corresponds to the task of classifying them into two groups—edible or poisonous—on the basis of a classification rule. To support this binary task, we have collected the largest and most comprehensive attribute based data available. In this work, we detail the creation, curation and simulation of a data set for binary classification. Thanks to natural language processing, the primary data are based on a text book for mushroom identification and contain 173 species from 23 families. While the secondary data comprise simulated or hypothetical entries that are structurally comparable to the 1987 data, it serves as pilot data for classification tasks. We evaluated different machine learning algorithms, namely, naive Bayes, logistic regression, and linear discriminant analysis (LDA), and random forests (RF). We found that the RF provided the best results with a five-fold Cross-Validation accuracy and F2-score of 1.0 ($\mu =1$, $\sigma =0$), respectively. The results of our pilot are conclusive and indicate that our data were not linearly separable. Unlike the 1987 data which showed good results using a linear decision boundary with the LDA. Our data set contains 23 families and is the largest available. We further provide a fully reproducible workflow and provide the data under the FAIR principles.

## Introduction

Mushrooms are available in a great variety. Commonly found in nature, the fungus is the visible fruiting body, while in the substrate there is an underground mycelium^1–3^. Fungi are responsible for breaking down waste and recycling the useful nutrients in the soil. They can be a delicious treat, part of a traditional meal or even have medicinal properties^4,5^. Since the discovery of penicillin, mushrooms have been the focus of many other discoveries^6^. From its use as a recycled organic resource^7^, to habitat-wide association with root-associated fungal communities in forests^8^, to fungal computers^9^.

Since most mushrooms found in nature appear to have common characteristics, there are many tips to identify edible mushrooms. However, different mushrooms may look very similar and can only be distinguished by one or two specific characteristics or attributes; e.g.  cap shape, gill color, odor, etc. Thus, deciding between edible and poisonous becomes a difficult task, let alone determining the species. In fact, many field guides and text books advise against using simple rules to determine edibility^2,3,10^. Due to this challenge, many applications and research works in different knowledge domains have been concerned with the identification and classification of mushrooms. Especially with the task of distinguishing poisonous from edible mushrooms.

There are two main approaches based either on image data or on the attributes mentioned above. The first is in computer vision research. Various machine learning techniques and algorithms are applied to a data set of mushroom images. The main idea is to classify mushroom images, without background, based on the features extracted from the image domain. In this specific knowledge domain, different variants of this approach and different algorithms have been studied. For example, neural networks (NN), support vector machines (SVM), decision trees or k-nearest neighbors (kNN)^11–13^. The second relates to attribute based research. Either motivated by the biological question of toxicity vs. edibility, or by evaluating novel algorithms and classifiers, attribute-based works relied on one specific mushroom data set^14–17^. The mushroom data set in question was provided by the University of California, Irvine (UCI) in 1987^18^. It includes descriptions of hypothetical entries corresponding to 23 species of gilled mushrooms in the Agaricus and Lepiota Family (pp. 500–525)^2^.

This data clearly distinguishes edible mushroom entries from poisonous ones, where poisonous includes unknown edibility and not recommended. Yet, it is too small and does not reflect the variety of mushrooms. In addition, it is used recurrently to demonstrate binary classification in education and as a use case for public understanding of applied machine learning. Although this data set is outdated in itself, it is still used today to show how ‘simple’ such a task is. This is in itself problematic because this data set is neither representative nor a good teaching example. Motivated by natural mushroom diversity and a more comprehensive data set, we describe the steps that lead to a new pilot data that includes a broader representation of mushroom species adapted for binary classification. For comparison purposes, we adopted the UCI 1987 data format to create such a data set. Our work makes five main contributions:

1. The creation of a data workflow to extract text book data entries, format these entries into a primary data set that contains the mushroom species, adopt variables from the nominal variables of the UCI 1987 data, and generate hypothetical mushroom entries as a secondary data using random sampling.
2. Since the newly created data follows the 1987 data format and encoding, comparability and benchmarking are possible. That is to say, the research papers that cite the 1987 data can be directly used on our pilot data.
3. A reproducible workflow to create, curate and simulate a pilot data for binary classification and evaluation. This includes the usage of four classifiers to address the task of binary classification and its evaluation by calculating multiple performance metrics and their interpretation.
4. The report of a suitable classifier capable of perfect classification results for the pilot data. The random forests classifier achieved perfect accuracy and F2 score metrics.
5. The creation of a more realistic data set that includes 173 mushroom species from 23 families and that is only non linearly separable.

## Results

**Figure 1:**
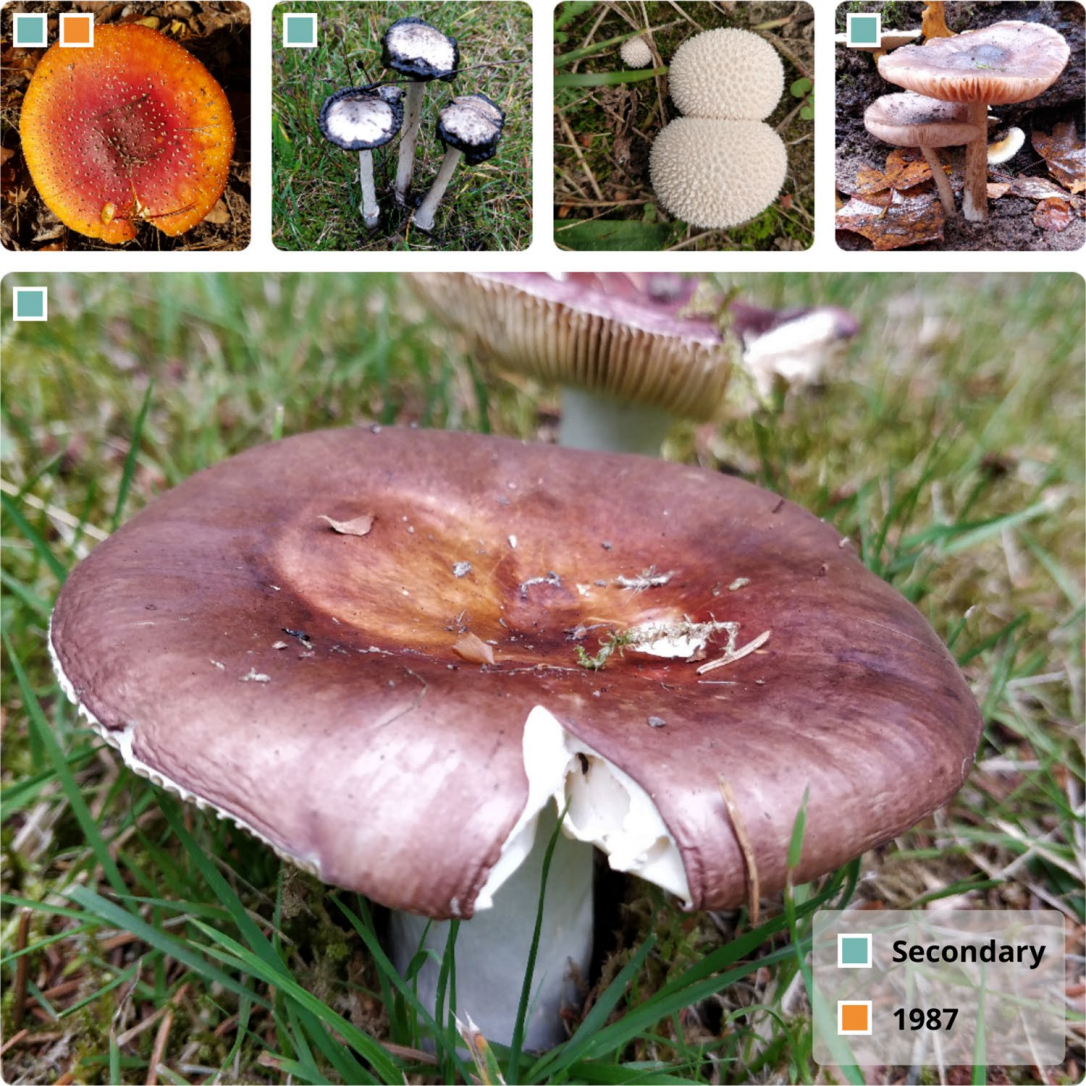
Annotated mushroom observations. From left to right, the annotated mushroom species are: *Amanita muscaria, Coprinopsis atramentaria, Pluteus cervinus*. The one image without an annotation corresponds to a species from the puffball mushroom family. Because stemless mushrooms species were excluded from the data, an identification cannot be made. The largest image is shown for a mushroom from the *Russula fragilis* species with the following attributes: sunken cap-shape, purple cap-color, whitegill-color, whitestem-color.

While the 1987 data is representative of 23 species from 2 closely related mushroom families, we have curated a total of 173 species from 23 mushroom families. This secondary data is simulated and is based on the third edition of the identification book guide *Mushrooms and Toadstools*^3^. It contains over 230 mushrooms and toadstools and rigorously describes information into attributes such as size, habitat, the season they can be found in, and whether they are edible and poisonous. In addition to the fact that many of the species are commonly found throughout the European continent, this subset makes the problem of creating a larger data set tractable. By including only stemmed mushrooms, the secondary data represents an approximate increase of 87% more species and 91% more families. Figure 1 depicts a handful of mushroom observations made in the course of summer/autumn of 2020 in Marburg, Germany. Since the 1987 data was created for only two families, the classification task will only be successful on the mushroom species that belong to these families. Thanks to the secondary data, we are able to classify additional mushroom species given the observed attributes.

To reproduce all data related operations and make our results transparent, we have shared our workflow. It contains all modules, scripts and all intermediary and final data sets and can be found at https://mushroom.mathematik.uni-marburg.de/. The data has also been assigned a digital object identifier (DOI)^19^. In addition, we report the primary data or secondary data as results in the next sections.

### Primary data

The primary data is saved in a comma separated file (CSV) and contains 24 columns with 3 classes and 21 variables. Table 1 reports the header with the first three entries as well as the last two entries.

**Table 1:** Primary data excerpt. The square brackets refer either to a set of nominal variables or to a continuous range of values. The single letters were encoded as nominal variables. Regarding continuous variables (seen as float numbers), they correspond to lengths in centimeters (cm), with the exception of *stem-width* reported in millimeter (mm). The shown columns are the three classes, the first two variables and the last variable. Intermediary columns are not shown due to page and column size restrictions.

Family	Name	Class	Cap-diameter	Cap-shape	Season
Amanita Family	Fly Agaric	p	[10:20]	[x, f]	[u, a, w]
Amanita Family	Panther Cap	p	[5:10]	[p, x]	[u, a]
Amanita Family	False Panther Cap	p	[10:15]	[x, f]	[u, a]
.	.	.	.	.	.
Morel Family	Common Morel	e	[3:8]	[p, c, o]	[s]
Jelly Discs Family	Jelly Babies	p	[1:1.5]	[x, f, s]	[u,a]

A subset of the variables had a large number of missing values, making them unusable. This was mainly due to the fact that the new source text book did not contain consistent information for all the mushroom attributes and the different species (c.f., Supplementary material)^3^. To remedy this problem, we contemplated the manual enrichment of the primary data by using a copy of the Enrich mushroom identification text book in the German language^10^. Although this would allow us to fill in the void for many species, such a process was deemed problematic due to a set of challenges. Aside from being time consuming and very demanding, enriching only a subset of the species could have led to major data inconsistencies. For these reasons, we did not enrich the primary data.

Although the designed modules were successfully parsed, extracted, and matched strings and sub-strings from each book entry, some mismatches occurred. The resulting primary data was manually curated and quality controlled.

### Secondary data

For the simulation, we chose to create 353 hypothetical mushrooms entries per species. This corresponds to the same number of entries per species in the 1987 data. Intermediary and final CSV files consisted of a header followed by the 61,069 hypothetical mushroom entries. The data comprised one binary class, 17 nominal variables and three quantitative variables. An excerpt of the final secondary data is reported in Table S2. The secondary data is to be considered a pilot as it is a simulated data set.

### Data quality and integrity

Both data sets were found to be balanced. The quality of balance is based on the *class* values: poisonous and edible. The overall ratio for the each data, 1987 and secondary data, was (*p* : 0.48, *e* : 0.52) and (*p* : 0.55, *e* : 0.45), respectively. Thanks to the coupling of random sampling and normal distributions, we successfully generated different mushroom entries. In Supplementary Fig. S2, we report an example of normal sampling of 500 values for each of the three qualitative variables; specifically for the mushroom species *Amanita muscaria* or Fly Agaric. The linear correlation of the normally sampled quantitative variables is then verified. An example visualization is reported in Fig. S3, where the linear correlation is visible in each plot.

**Figure 2:**
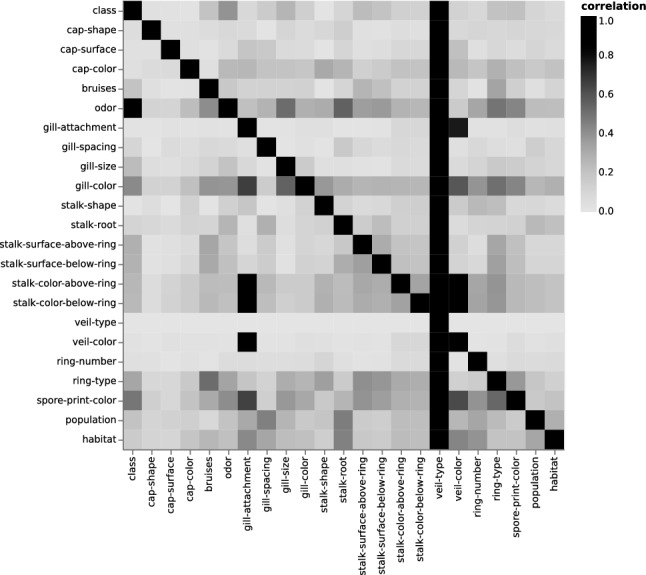
1987 data.

**Figure 3:**
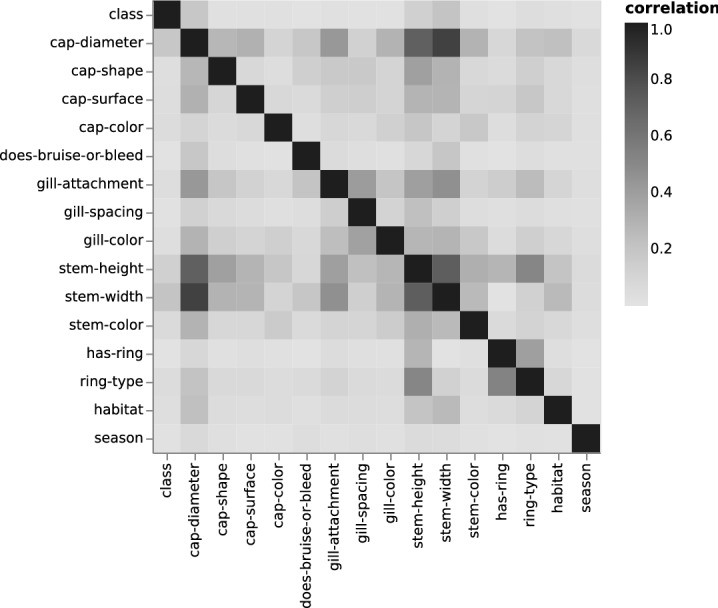
Secondary data.

To examine the correlation of the variables within each data set, we visualized all pairwise correlations as heat maps. Each heat map compares all the variables pairwise, which resulted into two variables at each cell position. Figures 2 and 3 depict the results for the 1987 and secondary data, respectively. The sequential gray scale palette is clipped so it encodes the highest correlation value of 1 in black and the lowest values in gray^20^. Depending on the considered variable pairs, symmetries and asymmetries were observed due to the different calculated correlations (e.g., Theil’s U, Person coefficient). For the heat map of the 1987 data, we observed that the *veil-type* is correlated with all the other variables, which makes it redundant. We noted multiple instances of such correlations. First, the correlation values for the *gill-attachment* with the *stalk-color* above and below the ring are both capped at 0.97. Second, the *stalk-color* highly correlated with the *gill-attachment* with the *veil-color* at 0.87 and 0.88, respectively. A third and last example is *odor*, which alone has a correlation value of 0.91 to *class*. This has made the classification task obsolete. Although this extremely high correlation is an outlier, about half of the variables have a *class* determining correlation between 0.25 and 0.5. In comparison, we found no *class* determining correlation greater than 0.2 for the secondary data. The only notable high correlations are the expected correlations for the continuous variables. These were determined by the assumed co-variance matrix as described in Eq. (1).

**Figure 4:**
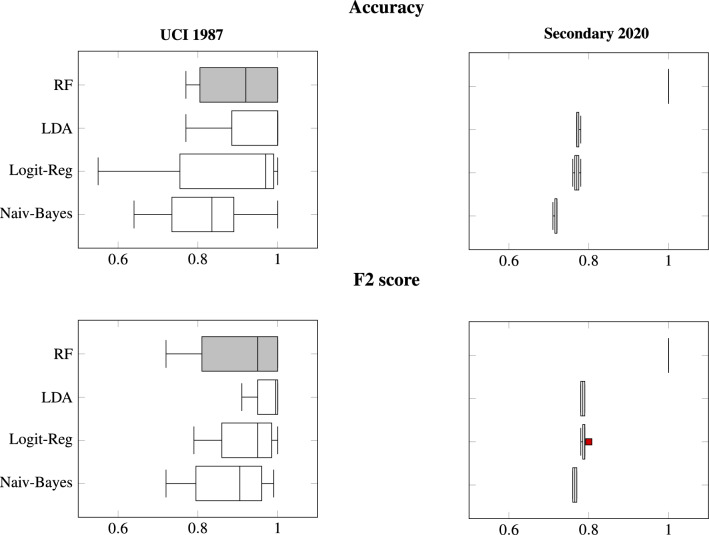
Five-fold cross-validation accuracy and F2 score results for both data sets and using each of the four classifiers. The highest score results are obtained using the RF classifier. They are reported in gray color.

### Evaluation

**Figure 5:**
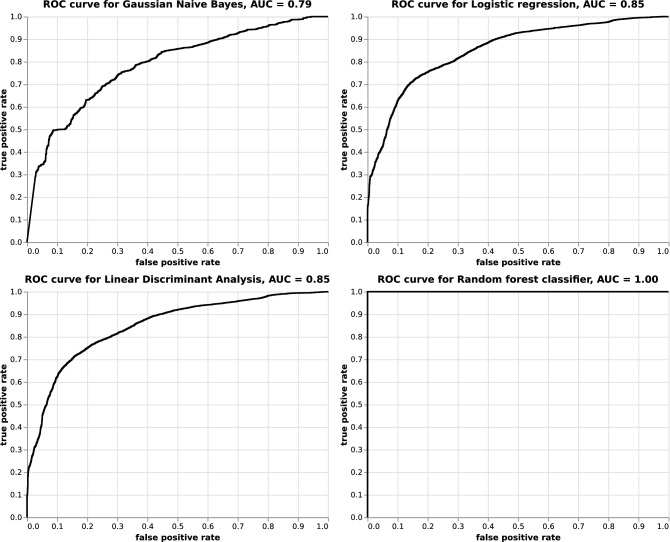
ROC curve for each classifier applied to the secondary data. The x-axis and the y-axis correspond to the FP and TP rates, respectively. The black line represents the ROC curve. The area under the curve represents the AUC which is reported textually above the graph. Each curve depicts the true positive rate or the recall on the y-axis and the false positive rate or the Type I error on the x-axis. The latter corresponds to the ratio of mushrooms wrongly classified as poisonous to all edible mushrooms.

We report the accuracy and F2 score results for each method or classifier in Fig. 4; from all trained models. In general, the RF was found to provide the best and most consistent results for the secondary data set. On the contrary, the LDA classifier was sufficient for successfully classifying the 1987 data, which points to a linear boundary separation in this data. As seen in the reported ROC curve of the RF in Fig. 5, the AUC on the secondary data is found to be optimal with a perfect classification score.

In terms of cross validation, the 1987 data performed significantly worse in cross-validation, while the secondary data had more consistent results independent of the used classifier. That is to say, the 1987 data showed a significantly higher variance, while the secondary data had no variance. These results were observed for both the accuracy and F2 score metrics.

Moreover, as seen in Fig. 4, similar accuracy and F2 score results were reported when using either the logistic regression or the LDA for the secondary data. When considering these classifiers, the accuracy of the secondary data was found to be good, but significantly worse than the accuracy of the 1987 data.

## Discussion

First, this work inscribes itself in putting a more substantial effort to creating a more comprehensive data set to addressing the task of binary classification for poisonous versus edible mushrooms. In its entirety, this work is easily reproducible. The methods are available as Python modules in the related repository and are described in the Supplementary material. Two data sets, namely the primary data and the secondary data were the main output of this work. Our classification results were conclusive. They especially showed that the 1987 data is linearly separable, while the secondary is only non linearly separable using RF. The historical 1987 data set is a product of simulation, neither the intermediary data was published, nor the employed methods to create the 8,124 hypothetical mushroom entries. For the new secondary data that we created, the intermediate variations of the data sets are accessible and easily reproducible. All source code and pertaining data available in the repository are open-source, freely available for modification and remixing under the Creative Commons License CC BY 4.0.

Second, the primary data is limited in its representation of reality. Aside from having many missing values and in its current state, the primary data makes no distinction between nominal values that occur alternatively and those that occur simultaneously. For the example occurrence of a simultaneous characteristic like the cap surface being shiny and sticky, random sampling from the set *{shiny, sticky}: {h, t}* is inappropriate. To account for this observation, the structure of the primary data would have to be reworked to distinguish between these two types of nominal values. This is possible by including subsets for simultaneous occurrences of mushroom characteristics.

Third, another limitation exists with the nominal values of the secondary data. This limitation addresses the fact that the simulation does not account for correlation between nominal variables. For example, the cap shape and surface of many mushroom species is convex/smooth when they are are younger and flattens/dries as they become older. Hence, mushrooms with the value *convex: x* for the variable *cap-shape* should usually have the value *smooth: s* for the variable *cap-surface*. To remedy this issue, a collaboration with mycologists is necessary to extend the primary data so to create entries of different stages of each mushroom species. For a more general approach, it is possible to incorporate the correlation among different characteristics by factoring it into the simulation process (nominal and quantitative). For the case of nominal data, this is complicated and it is even dangerous in the absence of empirically based correlations. For quantitative variables, an assumption is made for the correlation between the cap diameter, the stem height and the stem width. While the assumption that mushrooms with larger caps have longer and broader stems is logical, it does not hold true for all species.

Fourth, the fact that all fungal species share the same correlation represented by the assumption of a specific co-variance matrix may be misleading. However, our idea was to create a reproducible example where correlations are taken into account in the simulation process. To obtain experimentally based correlations, co-variances must be calculated from field observations, which can then replace the herein assumed co-variance matrix, c.f., Eq. (1). To further improve the realism, we would advise creating separate co-variance matrices for different mushroom species. Another couple of points are worth mentioning. One point is to create an even more diverse primary data set from larger text books. For example, we could recommend The Big Kosmos Mushroom Field Guide: All Edible Mushrooms with Their Poisonous Lookalikes from Kosmos Verlag as it includes over 1,200 mushrooms species^21^. Another point is to re-examine the creation of hypothetical entries by using other algorithmic approaches, for example interval regression to fit the interval nature of the primary data. Fifth, representing mushrooms as such low dimensional data points is quite challenging and a lot of simplifications and abstractions have to be made. To demonstrate this point, a good example are the color variables. For example, the *cap-color* with nominal values as *brown: n*, which is vague and ambiguous. A more precise and reliable approach could be RGB values or HEX codes. However, this would require gathering colors from pictures and considering for example the median color of an image region, such as the color of a mushroom cap. In addition, it would be worthwhile to establish a standard for the color coding, for example using the Munsell color system. Yet, it is important to note that most of the nominal variables present in the secondary data are still widely used and accepted.

Sixth, the secondary data inherits the data structure and format from the 1987 data. This is at best reductionist since this historical data only relied on observations of 23 species of gilled mushrooms from two closely related families. As a result, the structure only applies to mushrooms having a cap, gills and a stem. To conform to these constraints, 63 mushrooms were excluded from the new text book. While this illustrates the integration and reproducibility of our work, we would advise a broader and more flexible structure or data format to accommodate all variations of the different mushrooms and toadstools. Our findings indicated that although our data is not perfect, it is a much better and much more realistic alternative to the task of attribute based binary classification. In light of the current literature and its usage, our new pilot data should be considered as state of the art for the literature.

Seventh, we succeeded in obtaining reduced and fitting versions of the secondary data, to conform to the 1987 data format and to support the task of comparison.That is to say, the 1987 data showed a significantly higher variance, while the secondary data had no variance. It is worth noting that the identical number of variables opens the possibility of combining data sets created from different sources. One approach could explore classification when exchanging test sets between both data. Another approach is to merge the two data and analyze the resulting data set. While the matching method was good, creating such a match results in a loss of information due to the mutual exclusion of several variables. That is to say, making data versions with identical variables requires an exclusion of a lot of information. We deem it is not reasonable to run the classification to draw any conclusions. Generally, without using any of the previously mentioned approaches, results obtained using random forests are very good. Yet, it is important for future work to expand and enrich the primary data with more species. Moreover, multiple simulations and versions of a secondary data may be investigated by varying the co-variance matrix.

Eighth, the task of binary classification was explored to demonstrate a potential application of our pilot data for classification, the secondary data. We found that random forests were best suited to separate the data points present in this newly created data. This showed that our data are better at capturing the non linear nature of mushrooms. Cross validation results were stable and robust for all four classifiers on the secondary data. We may attribute it to the larger number of data points, it has about eight times more data points than the 1987 data. Our work sets a starting point for future benchmarking. Aside from using different classifiers, each classifier may also be adjusted to the problem. For instance, for all class predictions made in this work, the class dividing threshold was always chosen as 0.5. While this is a good starting point, the threshold may be fine-tuned to better adjust classification results.

Ninth and last, many mushrooms species are very similar to each other. A binary classification cannot be reliable. The primary data can be used to simulate other randomized versions of the data with an arbitrary number of hypothetical mushrooms per species. Indeed, since the primary data also featured the two multinomial classes *name* and *family*, it is also possible to simulate new variations of secondary data for multinomial classification. This means that instead of only identifying a mushroom as poisonous or edible, this work can be extended to identify a certain family or certain species. Furthermore, multivariate classification is also possible by simulating secondary data (with two or all three of the classes simultaneously). Moreover, we would suggest expanding on this work by not only looking at multinomial classification but also by approaching trained mycologists to safely verify the data. As a result, solving the problem of mushroom identification with text book based simulated data alone comes with many shortcomings. We believe it is really important to consider real life observations because they are a crucial foundation to all text books. With a more realistic starting point, the data may be extended with simulated data with the help of data augmentation. This would effectively provide the means to create more diverse data without adding new data points; in turn, circumventing the need for gathering and integrating more data.

## Methods

The methods is divided into seven data related tasks: formatting, extraction, curation, simulation, quality and integrity checks, binary classification and evaluation.

### Data format

The primary data follows the format or structure of the 1987 data; which is briefly detailed below. It is based on 23 species of gilled mushrooms from the book *The Audubon Society Field Guide to North American Mushrooms*^2^. Each nominal variable from the 23 variables of the 1987 is encoded with a single unique variable. It is reported as *category name: letter* on its first occurrence and from there on only as *category name*. The binary class *class* is separated into *edible: e* and *poisonous: p*. The *poisonous* subclass includes inedible mushrooms as well as those of unknown edibility. Further details of the data encodings are reported in Supplementary Table S1.

Motivated by the comparison of a new data to the historical 1987 data, we create the primary data by taking both the binary class and the names of the variables without changes. However, the possible values of the variables in the 1987 data are changed from single nominal values to sets of nominal values in the primary data. The possible nominal values and their 1-letter encoding remain unchanged. The values of the variables of the primary data are now reported as *{set of category names}: {set of letters}*. A possible value for the first variable of the primary data *cap-shape* is *{convex, flat}: {x, f}*. This corresponds to interpreting that the cap-shape of this mushroom species can be flat or convex. While each entry of the 1987 data represents a hypothetical mushroom entry, each entry in the primary data represents a mushroom species.

### Data extraction

The primary data is based on a total of 236 mushroom species from the third edition of the identification book *Mushrooms and Toadstools* from 2013^3^. Due to some species having either no cap, or with potentially missing attributes, 63 species were excluded. The book is separated into family groups, which are further divided into book entries for each species. A book entry has 6 structured parts:

1. the mushroom species name, in both English and Latin (including the family name, only if the given species is the first representative of a given family)
2. the general description in prose format containing information and characteristics (includes most of the variables)
3. the size attributes (incl. cap diameter, stem height and stem width),
4. the habitat (it is one variable in the primary data)
5. the season during which the species grows, and
6. the edibility or the binary class: poisonous vs. edible.

The data is extracted from the electronic publication or EPUB file format of the *Mushrooms and Toadstools* book^3^. This file format follows the Hypertext Markup Language or HTML, which is both human and machine readable. Since it defines a set of rules for encoding documents, we follow them to extract the data by using natural language processing modules. This includes parsing and extracting attribute based values from free form text in the Description of each mushroom species (Figure S1). All details modules and aids for running our scripts are reported in the Supplementary material.

As detailed in the previous section, each book entry has 6 structured parts. Each is extracted as follows:

1. the name is defined as a header and mapped to the same-named class. This header and thus the book entry is identified by the HTML $<p>$ tag which surrounds the name and has the *class* attribute value *“chapterHeadA”* (case insensitive). The string of characters defines that follows the $<p>$ tag with the *class* attribute value *“paraNoIndent”* contains one of the other structuring parts and defines the general description of a book entry.
2. the nominal variables are either present in the description text or are missing for the mushroom species found in the book entry. These variables exclude the *habitat* and the *season*. Sub-strings of the general description may correspond to the following variables: *cap*, *gills*, *veil*, *stem* and *ring*. This facilitates the mapping to the corresponding variable names by correctly matching sub-strings to the variable names. By following this logic, all positive matches are read out to the fitting nominal variables. For the example sentence *‘The entire young fruit body is enclosed in a white veil which leaves fragments (which may wash off) on the shiny red, marginally grooved cap.’*, the program extracts *{grooved, shiny}: {g, h}* for *cap-surface*, *{red}: {e}* for *cap-color*, *{universal}: {u}* for *veil-type*, and *{white}: {w}* for *veil-color*.
3. the three quantitative variables can be directly parsed from the size attributes.
4. the habitat is parsed and matched to its corresponding nominal variable by looking for the possible values in the subsequent sub-string after a positive match.
5. the same logic is employed for the season, and
6. the edibility is parsed to *class* since it always starts with either edible, inedible or poisonous.

### Data curation

For comparison purposes and due to the differences in the text book sources, the variables that describe the primary data are adapted to the historical data from 1987. We follow the aforementioned book entry structure to report the relevant changes.

1. the English *name* as well as the *family* are added as multinomial classes
2. the occurrences of *stalk* in variable names are replaced with *stem* since stem is more common in the text book. The variable *bruises?* is amended to *does-bruise-or-bleed* so to include latex bleeding mushrooms, which are absent from the previous 1987 data. The following variables are removed or changed since the information in the prose text is insufficient for these variables in their current form: *odor*, *gill-size*, *stem-shape* and *population*. The variables *stem-surface-above-ring* and *stem-surface-below-ring* as well as *stem-color-above-ring* and *stem-color-below-ring* are combined into *stem-surface* and *stem-color*, respectively. The combination is performed by not differentiating between above and below ring as this information is not present in the text. The variable *ring-number* is changed into *has-ring*
3. the variables *cap-diameter*, *stem-height* and *stem-width* are added as continuous quantitative variables since these are the size attributes (in appearing order) that are listed for almost all mushroom entries
4. the nominal variable *habitat* remains unchanged
5. the nominal variable *season* is added, and
6. the binary variable edibility remains unchanged.

The matched book entries are checked for inconsistencies to improve the data quality. This is a necessary step before any subsequent tasks.

### Data simulation

The secondary data are composed of hypothetical mushroom entries whose fitting characteristics are simulated from the primary data. To remain comparable to the 1987 data structure, we rely on single variable randomization. While the format of the secondary data is fully adapted from the primary data, the values of the variables move from nominal sets and quantitative ranges to single nominal and quantitative values, respectively. To address the simulation step, we consider the classes, the nominal variables, and the quantitative variables. The binary class *class* for edibility and the multinomial classes *name* and *family* have unambiguous values. Since they are adapted to the data format, they are carried over to the secondary data without any changes.

The nominal variables have nominal sets as values representing possible values for each mushroom characteristic. Starting from each nominal set, single nominal *n* values are randomly selected. While each set belongs to one mushroom species, every single value that is sampled belongs to a hypothetical mushroom. The simulation corresponds to a random sampling step, it is performed using the *choice* function. An additional simplification step is carried out to adapt the 1987 data format. For example, the *Fly Agaric* entry in the primary data has the value *{grooved, shiny}: {g, h}* for *cap-surface*. This is due to the corresponding book listing this species with a shiny and grooved cap. The simulation results in *n* hypothetical entries having either a grooved cap, or a shiny cap. In another example, the *red-brown* color that is listed in the book is interpreted as the value *{red, brown}: {e, n}*. This also leads to either red or brown mushroom entries.

The quantitative variables *cap-diameter*, *stem-height* and *stem-width* are continuous variables. These variables fall within a [*min*, *max*] interval and often associated with an average value or mean $\mu$. To reflect that, we create the following interval $[(1-\frac{1}{4})\mu , (1+\frac{1}{4})\mu ]$. In the case of stemless mushrooms, the variables *stem-height* and *stem-width* are set to 0. For the simulation step, the [*min*, *max*] intervals are respected. Multiple assumptions are fundamental to the herein presented methodology, which we describe below.

- The three variables: cap diameter, stem height, and stem width are normally distributed in each given interval. For each of those three variables, we rely on the standard normal distribution to generate *n* values from a normal distribution of *N*(0, 1), with $N(\mu ,\sigma )$.
- These three variables have a certain correlation. To improve the realism of the simulation step, an explicit correlation to their normal distributions can be added. For an empirical result, the co-variances should be calculated from field observations. To demonstrate our method, we assume the following co-variance matrix, where the stem related quantities correlate somewhat stronger.
  1 $$\begin{aligned} COV=\begin{bmatrix} 1 &{} 0.5 &{} 0.5 \\ 0.5 &{} 1 &{} 0.7 \\ 0.5 &{} 0.7 &{} 1 \\ \end{bmatrix} \end{aligned}$$
  with the variance of each normal distribution to be $\sigma ^{2} = 1$ and the co-variances between cap diameter (a), stem height (b), and/or stem width (c):
  2 $$\begin{aligned} COV_{(a,b)}=COV_{(a,c)}=0.5, \, COV{(b,c)}=0.7 \end{aligned}$$
- To generate three correlated normal samples from the three uncorrelated normal samples, a matrix decomposition is calculated as $COV=LL^{\top }$^22^. Since a co-variance matrix is always positive semi-definite, that is to say all eigenvalues of the co-variance matrix are non-negative^23^, *L* can be obtained as a lower triangular Cholesky decomposition. To obtain the final samples, we multiply each of the three uncorrelated normal samples with *L*, the resulting matrix:
  3 $$\begin{aligned} L=\begin{bmatrix} 1 &{} 0 &{} 0 \\ 0.5 &{} 0.87 &{} 0 \\ 0.5 &{} 0.52 &{} 0.69 \\ \end{bmatrix} \end{aligned}$$
- The resulting normally distributed values of *n* for each variable can each be assumed as hypothetical mushroom entries. For each variable, we assign a correlated normal sample of size *n*. The sample is resized by calculating $x = \frac{1}{2}(x + 1)$ for each value, resulting in a normal distribution with $\mu =0.5$. Then $x=x*(max - min)+min$, so $99.7\%$ of the normal sampled values fall within the range of the interval [*min*, *max*].

### Data quality and integrity

Apart from the previously covered data curation step of the primary data, it is important to ensure the data quality and integrity. We briefly describe quality checks and preprocessing steps. Further details are reported in the Supplementary Material.

- *Balance and comparability* of the secondary data and the 1987 data. The balance of the data is examined by analyzing the occurrence of poisonous vs. edible (nominal variable *class*). The ratio of the missing values for each variable and the auto-correlations among each variable pair. The latter depends on the variable type (e.g., Theil’s U, Pearson correlation of a quantitative variable pair)^24,25^. Variable pair correlations are visualized as a heat map as seen in Figures 2 and 3.
- *Second data curation* for correct data encoding. We handle missing values by using an imputation method, that is to say a threshold based filtering. First and to limit the dilution of the analysis, variables with more then 50% missing values are removed. Second, the remaining data with missing values are replaced using the most frequent single imputation^26,27^.
- *Data transformation* for machine learning. The data are split into training and testing sets for the binary classification task. It is imperative to explicitly encode all values in both data into numerical values. This is due to the classifiers implementation which only accepts numerical values as input. The binary class is label encoded using *sklearn*’s functionality. For instance, the value *poisonous: p* is encoded as 1 and *edible: e* as 0. The quantitative variables remain unchanged, while the nominal variables are one-hot encoded.
- *Direct data mapping* as alternative comparison. This mapping between the two data sets – 1987 data and secondary data – is performed by means of duplicating matching names and merging them in the same row of the CSV file. On the contrary, for a variable with no apparent match, two alternatives are possible and this is encoded as empty string. Indeed, a variable with the corresponding name is either added to the other data containing the value 0 (no mushroom entry has a corresponding nominal value), or the variable is renamed into a pre-existing variable.
- *Training and testing data* sets are prepared using the standard Pareto principle. The training set is randomly sampled without substitution, it represents 80% of the data. The remaining 20% are used as a test set. To further assess the predictive performance of the machine learning models on the data, a five-fold cross-validation is employed.

### Binary classification

The predictive models are created with the help of five different classifiers: Naive Bayes, logistic regression, linear discriminant analysis, and random forests. We briefly describe the assumptions and benefits of each method.

- Naive Bayes is a set of classifiers for supervised learning that use the Bayesian theorem^28^. The data is assumed to be pairwise independent and the data ought to be balanced.
- The logistic regression is a statistical model popularly used as a supervised learning classifier^29^. It assumes independence of errors, linearity in the logit for continuous variables, absence of multi-collinearity, and lack of strongly influential outliers.
- The Linear Discriminant Analysis (LDA) is a generalization of Fisher’s linear discriminant, it finds a linear combination of features that characterizes or separates two or more classes^30^. It makes the assumption such as the explanatory or predictor variables must be normally distributed.
- Random forests (RF) classifier is a supervised learning algorithm. The forest it builds, is an ensemble of decision trees. This ensemble of multiple decision trees are merged together to get a more accurate and stable prediction. RF have no formal distribution assumptions. They are non-parametric and can handle skewed and/or multi-modal data, as well as categorical data that are ordinal or non-ordinal.

### Evaluation

First, predictions are made using the aforementioned four classifiers. The outcome corresponds to obtaining a probability of a mushroom belonging to the class *poisonous: p* of each listed entry. To convert the probabilities into class predictions, a threshold is chosen. Without further information, we maintain the standard approach of the commonly used threshold of 0.5. This results in a mushroom being classified as *poisonous: p* if the probability is greater or equal to 0.5 and as *edible: e* if it is less than 0.5.

Second, we report different scoring metrics and the ROC curve to show the model performance. On one hand, different classical metrics are derived from the confusion matrix^31^: accuracy, precision, recall. We add to this list the *F beta score* which balances the recall and the precision metrics by calculating a weighted harmonic mean. To avoid false negatives (FN), we specifically add the F2 score by giving twice as much importance to recall as to precision. On the other hand, we report graphical representations of each model performance as ROC plots in Fig. 5. Unlike the aforementioned metrics, which are derived from the number of predicted classes, the ROC curve looks at the prediction probabilities before assigning a class dividing threshold.

Third and last, cross-validation is performed to create *k* separate evaluations. It permitted us to show how well a classifier performs with different training set choices. Since multiple evaluations are performed, the mean and variance of the recall and the F2 score are calculated and reported.

## Conclusion

The newly created mushroom data, mentioned herein as the secondary data, did a better job at capturing the complexity of mushroom identification than its predecessor, the 1987 data. Although it is a pilot data for classification task, this new data set required a classifier capable of separating data points using a non linear boundary. Indeed, the random forests classifier achieved nearly perfect results.

Our work encompasses a new secondary data set that passes data quality and integrity checks as well as a workflow that enables the creation of such pilot data from curated primary data. For example, the correlation between variables in the secondary data in Fig. 3 was a testament to that. While several variables are strongly determined by other variables in the 1987 data, this was not the case for our pilot data. Moreover, the higher correlations were expected as they are determined by the co-variance matrix. Taken together, our work makes the problem of creating not only larger data sets tractable but also adjusting for growth rates of specific mushroom families.

Since the 1987 data looks at a very specific subset of mushrooms, that is to say, 23 species from only two families, it led to an over-simplification of the general task of mushroom classification. This point is especially important when interpreting the binary classification results. Moreover, the cross-validation results were significantly worse for the 1987 data. The results for the 1987 data had significantly higher variance for every single classifier, while the secondary data proved to be robust with little to no variance. The best binary classification results were obtained with LDA for the 1987 data and RF for the secondary data, respectively. This was indicative of the linear separability of the data points in the historical data set but not for the new proposed data set.

Although the secondary data set serves as a pilot data for classification tasks, perfect classification results indicate that it is a better alternative to the 1987 data. Moreover, our results show that our data set is more suitable in terms of data quality, data integrity, robustness in cross validation, and in its representation of mushroom species. Withal, even if it does not contain all known mushroom species it presents a more realistic data set and should be adopted as it is a better alternative. We believe our approach provides a stepping stone for the research community to create and use better attribute based mushroom data sets and for the public understanding of machine learning approaches.

## Supplementary Information

Supplementary Information
