# Supplementary material for: Mushroom data creation, curation, and simulation to support classification tasks
Source: Sci Rep. 2021 Apr 14;11:8134. doi: 10.1038/s41598-021-87602-3 (PMC8046754; doi:10.1038/s41598-021-87602-3)
Supplement: Supplementary file 1 — Supplementary Information [file 41598_2021_87602_MOESM1_ESM.pdf]

Mushroom data creation, curation, and simulation to support classification tasks

Wagner et *al*, 2021.

Supplementary material

class	cap-shape	cap-surface	cap-color	...	population	habitat
p	x	s	n	...	s	u
e	x	s	y	...	n	g
e	b	s	w	...	n	m
...	...	...	...	...	...	...
p	k	y	n	...	v	l
e	x	s	n	...	c	l

Table S1: The structure of the 1987 data.

class	cap-diameter	cap-shape	cap-surface	...	habitat	season
e	6.20	x	h	...	d	u
e	8.96	f		...	d	u
p	2.27	f		...	d	u
...	...	...	...	...	...	...
p	3.32	f	y	...	d	a
e	3.28	s		...	d	a

Table S2: The structure of the secondary data excerpt. The table shows the header (bold text), the first 3 and the last 2 entries (from top to bottom), respectively. The shown columns are the binary class, the first quantitative variable and nominal variables. The single letters were encoded nominal values. As described in the primary data, the continuous variables are lengths in cm, except for the *stem-width* (mm). While, the empty cells represent missing values, the three dots represent skipped columns and values for presentation purposes.

```
Russulaceae Family,

Fragil Brittlegill,

Russula fragilis,

(DESCRIPTION)
While all Brittlegills are crumbly this small
species is especially delicate, as its name
implies. The thin-fleshed, flat cap is often
depressed at the centre where it is a darker
purple in contrast to the more violet or pink
margin, which is usually grooved. The pale
-cream, adnate gills have toothed edges
(visible only through a lens). The white
stem is tinged with yellow and is swollen
at the base. It smells of boiled sweets
(pear drops),

SIZE Cap 2-6 cm, stem 3-6 cm x 5-10 mm,

HABITAT Usually under birches but also
with beech or oak,

SEASON Late summer to autumn. INEDIBLE
has a very acrid taste,

SIMILAR SPECIES Birch Brittlegill (p.80)
is more yellow-pink, has more widely
spaced white gills and lacks the fruity smell,
```

Figure S1: Example of a mushroom species from an actual textbook entry (book form). The mushroom species is *Russula fragilis*. Natural Language Processing helped parse and extract attribute based information from free form text (Description) and from specific attribute names (size, habitat, etc) to create the primary data.

Data creation

Missing values

- The process of data creation and curation led to consistent results, however many missing values were reported.
- For the 1987 data, from 22 variables only 1 variable had missing values: *stalk-root*: 30,5%.
- For the secondary data, from 21 variables 9 variables had missing values. We report these variables below by descending order in percentage (%): *veil-type*: 94.8, *spore-print-color*: 89,6, *veil-color*: 87.9, *stem-root* 84.4, *stem-surface*: 62.4, *gill-spacing*: 41, *cap-surface*: 23.1, *gill-attachment*: 16.2, *ring-type*: 0.04. We noted that all variables with missing values were nominal ones.
- All missing values were marked as empty strings (secondary data) and as question marks or *missing* (1987 data).

Data quality and integrity

- **Balance and comparability** of the secondary data and the 1987 data. For the asymmetrical correlation of two nominal variables, the Theil's U or the uncertainty coefficient is calculated. For a given variable pair, an asymmetrical correlation is interpreted as how much information the first variable provides to the second. For the symmetrical correlation of a nominal and a quantitative variable, the correlation ratio is calculated. For the symmetrical correlation of two quantitative variables, the Pearson correlation coefficient is calculated.
- **Second data curation** for correct data encoding. We handle missing values by using an imputation method, i.e., a threshold based filtering. Replacements using the most frequent single imputation is a strong simplification, however it is a common approach for missing imputation of nominal variables.
- **Data transformation** for machine learning. The nominal variables are one-hot-encoded. This assumes that there exists for each possible value of a nominal variable a separate binary variable. Indeed, this step extends the name of the nominal variable with the original nominal value, where each nominal variable is encoded as a vector of the size of its possible different nominal values. This increased the subsequent number of variables, i.e., from 20 to 119.
- **Direct data mapping** as alternative comparison. These data manipulations are possible thanks to two intermediary 1-column CSV files with the encoded variable names as values for each of the two data. For instance, the variable *cap-color* from the 1987 data has the value *cinnamon: c* while the secondary data lacks this value, simply because cinnamon does not appear in the new source. In this case, the encoded variable *cap-color-c* is

renamed to *cap-color-n* representing the shade of brown or the nominal value *brown: n*.

- **Training and testing data** sets are prepared using the standard Pareto principle. The data is divided into equal 5 folds. Then, over 5 iterations, each of the 5 folds are used once as a test set, while the remaining 4 folds are used as a training set. At each iteration, the predictive performance is evaluated. This leads to results that do not only depend on the choice of a specific training set.

Aid for running the scripts

Three modules are implemented to handle the parsing of the electronic publication and the matching of sub-strings to target variables.

- The `data-cat` module contains file paths and dictionaries for the nominal variables
- The `text-attr-match` module contains utility functions to parse the textbook
- The `primary-data-gen` module calls the aforementioned modules and handles the parsing procedure to output a preliminary primary data as a comma-separated values or CSV file. This procedure creates a corresponding entry to each mushroom species or book entry
- The `gen-corr-norm` contains functions for generating the correlated normal distributions, which are used for the simulation step and for the visualization of both the distribution and the correlation plots. To simulate new randomized secondary data, the binary class from the primary data and randomly selected values for the variables are considered
- The `secondary-data-gen` handles input/output calls and simulates the secondary data. The result of the simulation has two outputs. While a CSV file is saved with the same order as the mushroom species of the primary data, each line is randomly sampled without substitution and the result is written to another final CSV file.

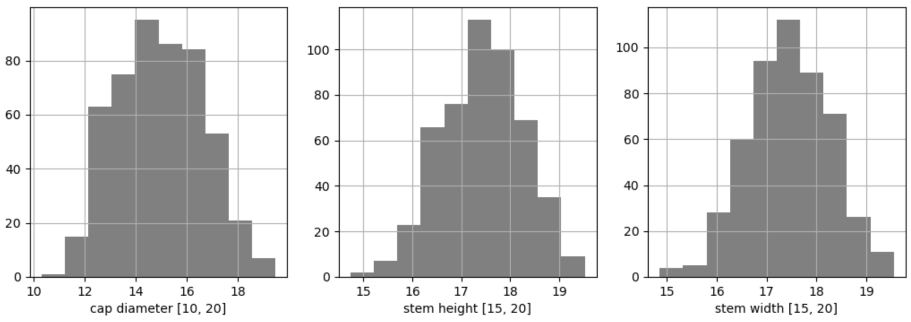

Figure S2: Bar plots for *cap-diameter*, *stem-height* and *stem-width* (left to right). The x-axis represents one of the variables, while the y-axis represents the frequency for each value. Note that the y-axes are only identical for the middle and right distributions. Results are plotted for a normal sampling of 500 values for each of the 3 qualitative variables specifically for the mushroom species *Amanita muscaria* or Fly Agaric.

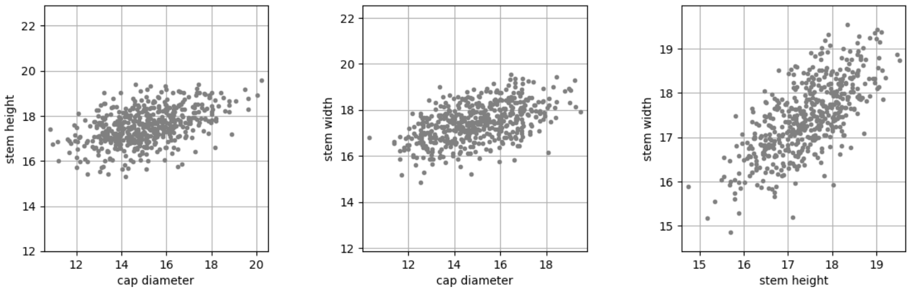

Figure S3: To verify the linear correlation of the normally sampled quantitative variables, we visualized the scatter plots of the resulting 500 values. Each plot corresponds to 1 out of 3 possible pairwise combinations among the 3 variables: *cap-diameter*, *stem-height* and *stem-width*. We found that the linear correlation is visible in each plot.

UCI 1987		Secondary 2020	
name	value	name	value
odor_n	0.138231	stem-width	0.101355
gill-size_b	0.061669	stem-height	0.071043
odor_f	0.057234	cap-diameter	0.065044
gill-size_n	0.054663	stem-color_w	0.038309
spore-print-color_h	0.050448	gill-color_w	0.025364
stalk-surface-below-ring_k	0.047932	gill-spacing_c	0.024314
stalk-surface-above-ring_k	0.043002	gill-attachment_p	0.022307
bruises_t	0.035813	gill-spacing_d	0.021634
ring-type_p	0.034615	does-bruise-or-bleed_f	0.019434
gill-color_b	0.032991	gill-attachment_a	0.018826
population_v	0.025939	does-bruise-or-bleed_t	0.018178
bruises_f	0.024332	gill-attachment_x	0.017792
ring-type_l	0.021561	cap-surface_t	0.017250
stalk-root_e	0.018477	has-ring_t	0.016125
spore-print-color_k	0.017560	cap-shape_x	0.015593

Table S3: Feature importance of the variables for each data set. Only the TOP 15 are shown. In terms of feature importance, the stem and cap based variables appearing as TOP 3, in the secondary 2020 data, do not figure in the TOP 15 of the UCI 1987 data.
